# Adolescents exhibit reduced Pavlovian biases on instrumental learning

**DOI:** 10.1038/s41598-020-72628-w

**Published:** 2020-09-25

**Authors:** Hillary A. Raab, Catherine A. Hartley

**Affiliations:** 1 Department of Psychology, New York University, New York, NY USA; 2 Center for Neural Science, New York University, New York, NY USA

**Keywords:** Human behaviour, Motivation, Learning and memory, Learning algorithms

## Abstract

Multiple learning systems allow individuals to flexibly respond to opportunities and challenges present in the environment. An evolutionarily conserved “Pavlovian” learning mechanism couples valence and action, promoting a tendency to approach cues associated with reward and to inhibit action in the face of anticipated punishment. Although this default response system may be adaptive, these hard-wired reactions can hinder the ability to learn flexible “instrumental” actions in pursuit of a goal. Such constraints on behavioral flexibility have been studied extensively in adults. However, the extent to which these valence-specific response tendencies bias instrumental learning across development remains poorly characterized. Here, we show that while Pavlovian response biases constrain flexible action learning in children and adults, these biases are attenuated in adolescents. This adolescent-specific reduction in Pavlovian bias may promote unbiased exploration of approach and avoidance responses, facilitating the discovery of rewarding behavior in the many novel contexts that adolescents encounter.

## Introduction

From an early age, individuals can rely on distinct forms of learning to maximize rewards and avoid punishments in their environments. Through Pavlovian learning, a neutral cue that predicts reward or punishment acquires the positive or negative value associated with its outcome^1^. This learned association between a cue and its predicted outcome can then drive reflexive behavioral responses that are elicited in a valence-dependent manner. Expectations of reward typically lead to approach behaviors^2,3^, whereas expectations of punishment tend to foster the inhibition of action^4^. For instance, by learning that a sweet smell often precedes enjoying a scrumptious dessert, you might be drawn toward the fragrant scent in anticipation of an afternoon treat. Or by learning that the sound of a horn often is heard before a car accident, you might freeze upon hearing the noise in anticipation of a negative event. This evolutionarily conserved learning mechanism allows approach or action inhibition to be readily deployed as “default” reactions to anticipated rewards or threats, respectively, without any need to evaluate the efficacy of these responses through experience^5^. Whereas Pavlovian learning couples expectations of reward or punishment with reflexive reactions that have no causal influence on the outcomes that actually occur, instrumental learning enables the discovery and deployment of actions that can directly influence the likelihood of obtaining reward or avoiding punishment. By learning which actions bring about beneficial outcomes, an individual can flexibly engage active or inactive responses based on their causal efficacy, rather than emitting reactive responses that are controlled by cues in their environment.

Pavlovian and instrumental learning systems do not operate in isolation^6^. When active approach responses are required to secure reward, or one must inhibit action to avoid punishment, the Pavlovian reactions aligned with these instrumental contingencies can facilitate learning. However, when Pavlovian tendencies conflict with instrumental contingencies, these default reactions can impede the learning of adaptive instrumental actions. For example, freezing at the sound of a car horn might be beneficial if you are about to step into oncoming traffic, but might be harmful if it prevents you from running out of the way if you are already in the road. Studies across species highlight the difficulty of learning to perform instrumental actions that conflict with Pavlovian expectancies, as the evolutionarily conserved tendencies to approach cues predictive of reward or to inhibit action to cues predictive of punishment can be too robust to overcome^7–12^. In active avoidance paradigms, rodents have difficulty learning to cross to the opposite side of a conditioning chamber in order to avoid being shocked, due to their tendency to freeze following a threat-predictive cue^13–15^. Similarly, in the appetitive domain, rats have difficulty learning to earn a food reward by not approaching a cue that predicts the reward^16^. These studies demonstrate that Pavlovian reactions to anticipated reward or punishment can pose constraints on instrumental action, disrupting flexible goal-directed learning.

To date, the biasing effects of Pavlovian learning on behavior have been studied primarily in adult rodents and humans^10,17–23^. While both Pavlovian and instrumental learning are evident from early childhood^24–27^, relatively few studies have investigated the interaction between Pavlovian and instrumental learning across development in humans^28^. Thus, it is unclear when Pavlovian constraints on action learning emerge and how they change from childhood to adulthood.

**Figure 1:**
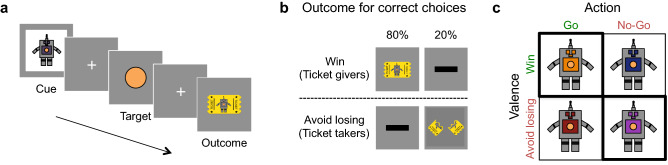
Task assessing Pavlovian influences on instrumental learning. (**a**) On each trial, participants saw one of four distinctly colored robots (cue). Participants could then either press (“Go”) or not press (“No-Go”) the robot’s “button” (the target) when it appeared. (**b**) Following their choice, participants received probabilistic feedback (outcomes for “Win” trials: win a ticket or neither win nor lose a ticket; outcomes for “Avoid Losing” trials: neither win nor lose a ticket or lose a ticket). (**c**) Each uniquely colored robot, which corresponded to one of the four trial types, was associated with a correct response (“Go” or “No-Go”) and an outcome (rewards or punishments). Pavlovian reactions and instrumental contingencies were aligned for the trial types on the bolded diagonal, whereas for the other two trial types they were in opposition.

In the present study, we tested the extent to which Pavlovian reactions differentially biased instrumental learning across development. We hypothesized that these Pavlovian learning constraints would decrease from childhood to adulthood, as the cognitive capabilities and neural circuits that support goal-directed instrumental learning are refined^25,29–32^. To test this hypothesis, we had 61 participants, 8–25 years of age (20 children aged 8–12, 20 adolescents aged 13–17, and 21 adults aged 18–25; see methods for detailed demographics of participants), complete a probabilistic Go/No-Go task in which valence and action were orthogonalized. We adapted a well-validated paradigm^20,21,33^ for use in a developmental cohort by using a child-friendly narrative to frame the task. The goal of the task was to earn as many “tickets” as possible by interacting with four different colored robots. Valence (i.e., the potential to either win or lose a ticket) and action (i.e., the need to either press or not press a button) were orthogonalized across the four robots. Two of the robots were associated with the potential to win a ticket (“ticket givers”), and two robots were associated with the potential loss of a ticket (“ticket takers;” Fig. 1). For both “ticket givers” and “ticket takers,” the correct response for one of the robots was to press a button, whereas the correct response for the other robot was to withhold a button press. Participants were instructed that robots could be either “ticket givers” or “ticket takers” and that the correct action for each robot could be learned through feedback. For the “ticket givers,” a correct response resulted in winning a ticket 80% of the time but no ticket (the “null” outcome) 20% of the time; whereas for the “ticket takers,” a correct response avoided the loss of a ticket (the “null” outcome) 80% of the time but resulted in the loss of a ticket 20% of the time. Incorrect responses yielded feedback with reversed outcome probabilities.

The Pavlovian default responses to approach expected reward or to inhibit action in the face of potential punishment were aligned with the correct instrumental response on “Go to Win” and “No-Go to Avoid Losing” trials, whereas the default responses were in conflict with the optimal instrumental actions on “Go to Avoid Losing” and “No-Go to Win” trials. Critically, a Pavlovian bias was evident if performance was better for the robots for which Pavlovian tendencies and correct instrumental responses were congruent than those for which they were incongruent. If Pavlovian learning did not bias instrumental action learning, then performance would be comparable across all four trial types.

## Results

To characterize patterns of age-related change in task performance, we assessed through model comparison whether each measure of choice behavior was best captured by a statistical model that included age alone (i.e., a linear model) or a model that included an additional nonlinear age-squared term, as in previous studies^34–36^. The best-fitting model thus indicates whether the developmental trajectory of choice behavior exhibits a continuous linear progression from childhood to adulthood, or whether behavior shows either adolescent-specific effects (i.e., children’s and adults’ behavior are more similar to each other than to adolescents’ behavior) or adolescent-emergent effects (i.e., behavior of adolescents is more similar to adults’ behavior than to children’s). Age and age-squared were included as continuous variables in all analyses. However, age is represented categorically (i.e., grouping children, adolescents, and adults) in some figures for the purpose of depicting results, and we use categorical age terminology in our interpretation and discussion of the findings.

### Behavioral analyses

**Figure 2:**
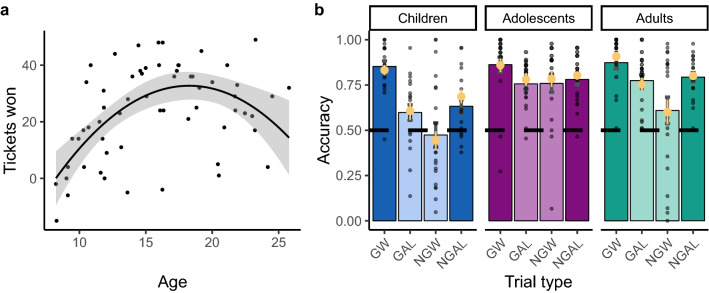
Behavioral performance by age. (**a**) Number of tickets won is plotted as a function of age. A quadratic line of best fit is shown. The error bars represent a .95 confidence interval. (**b**) Mean accuracy across all trials is plotted for each trial type, separately by age group. The darker shaded bars depict the trials for which Pavlovian tendencies are aligned with the optimal instrumental response, and the lighter shaded bars depict the trials for which Pavlovian tendencies are in conflict with the optimal instrumental response. Yellow points represent mean accuracy that was calculated from simulating data using the parameter estimates for each participant from the best-fitting model. The following abbreviations are used: GW: Go to Win; GAL: Go to Avoid Losing; NGW: No-Go to Win; NGAL: No-Go to Avoid Losing. Error bars represent ± 1 SEM.

First, we tested whether there was a relationship between the number of tickets won and participant age. A linear model that included both age and age-squared as predictors of number of tickets won significantly improved the fit compared to a model that included age alone (*F*(1,58) = 15.959, *p* = 0.0002). We found significant effects of age (*β* value = 7.523, s.e. = 1.804, *t*(58) = 4.170, *p* < 0.001, Cohen’s *ƒ*^2^ = 0.300) and age-squared (*β* value = −7.334, s.e. = 1.836, *t*(58) = -3.995, *p* < 0.001, Cohen’s *ƒ*^2^ = 0.275) on the number of tickets won, indicating a peak in overall task performance during late adolescence (Fig. 2a).

We next examined how participants’ choice behavior gave rise to this nonlinear age-related change in task performance. The qualitative pattern of performance on each trial type is depicted for children, adolescents, and adults in Fig. 2b (and Supplementary Fig. S1). To test how performance for each trial type differs as a function of age, we performed four separate linear regressions in which age and age-squared predict accuracy. The model that included age alone provided the best fit for accuracy on “Go to Win” (GW) trials, although age was not a significant predictor (*F*(1,58) = 0.557, *p* = 0.458; age: *β* value = 0.013, s.e. = 0.020, *t*(58) = 0.647, *p* = 0.52, Cohen’s *ƒ*^2^ = 0.007. For all other trial types (i.e., Go to Avoid Losing (GAL), No-Go to Win (NGW), No-Go to Avoid Losing (NGAL)), the model that included an age-squared term provided a better fit than the model with age alone (GAL: *F*(1,58) = 9.937, *p* = 0.003; NGW: *F*(1,58) = 8.828, *p* = 0.004; NGAL: *F*(1,58) = 10.004, *p* = 0.002). For these three trial types, age and age-squared were significant predictors of accuracy (GAL age: *β* value = 0.089, s.e. = 0.020, *t*(58) = 4.447, *p* < 0.001, Cohen’s *ƒ*^2^ = 0.341; GAL age-squared: *β* value = − 0.064, s.e. = 0.020, *t*(58) = − 3.152, *p* = 0.003, Cohen’s *ƒ*^2^ = 0.171; NGW age: *β* value = 0.084, s.e. = 0.038, *t*(58) = 2.204, *p* = 0.032, Cohen’s *ƒ*^2^ = 0.084; NGW age-squared: *β* value =  − 0.116, s.e. = 0.039, *t*(58) =  − 2.971, *p* = 0.004, Cohen’s *ƒ*^2^ = 0.152; NGAL age: *β* value = 0.080, s.e. = 0.018, *t*(58) = 4.402, *p* < 0.001, Cohen’s *ƒ*^2^ = 0.334; NGAL age-squared: *β* value = − 0.058, s.e. = 0.018, *t*(58) = − 3.163, *p* = 0.002, Cohen’s *ƒ*^2^ = 0.172). Thus, apart from GW for which accuracy was the highest and was comparable across age, performance on the other three trial types exhibited significant nonlinear improvements from childhood into adulthood.

**Figure 3:**
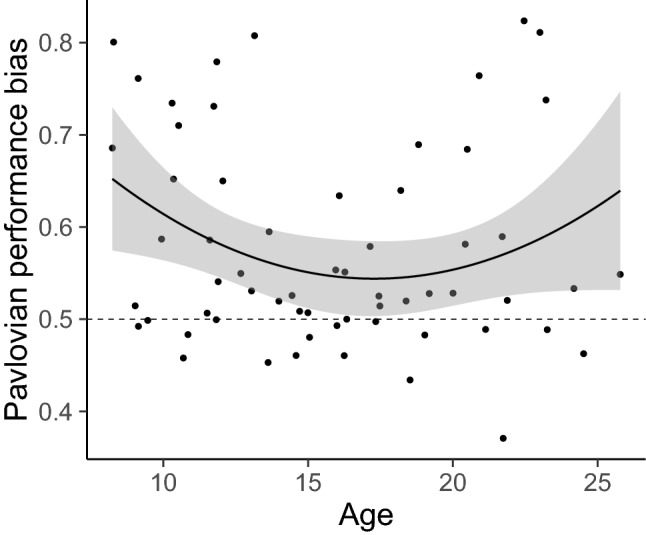
Pavlovian performance bias score by age. A performance bias of .5 indicates no Pavlovian bias, whereas larger scores represent greater Pavlovian interference with instrumental learning. The relationship between age and Pavlovian bias score is best fit by a quadratic function. The error bars represent a .95 confidence interval.

To quantify the Pavlovian influence on instrumental learning for each individual, we first calculated a Pavlovian performance bias score by averaging how often reward-related cues invigorated action (number of Go responses to Win cues/total number of Go responses) and how often punishment-related cues suppressed action (number of No-Go responses to Avoid Losing cues/total number of No-Go responses). A bias score of 0.5 indicates the absence of a Pavlovian bias; whereas higher scores reflect a greater Pavlovian bias on action, with 1 being the maximum bias score. The best-fitting linear model predicting age-related changes in this bias score included both age and age-squared as regressors, of which age-squared was a significant predictor of Pavlovian performance bias (*F*(1,58) = 4.136, *p* = 0.047; age: *β* value = −0.018, s.e. = 0.014, *t*(58) = − 1.271, *p* = 0.209, Cohen’s *ƒ*^2^ = 0.028; age-squared: *β* value = 0.03, s.e. = 0.015, *t*(58) = 2.034, *p* = 0.047, Cohen’s *ƒ*^2^ = 0.071). This analysis revealed that children and adults exhibited a greater Pavlovian bias than adolescents (Fig. 3). This bias appears to be driven comparably by both a reward-driven invigoration of action and a punishment-driven suppression of action (Supplementary Figure S2). A mixed-effects logistic regression corroborated this adolescent-specific attenuation of Pavlovian bias on learning (Supplementary Table S1).

### Computational modeling

By formalizing the value computations involved in learning from valenced outcomes, computational models can disentangle the contribution of a Pavlovian bias on choice behavior from differences in value updating, choice stochasticity, a value-independent bias toward action, or sensitivity to reward. We used reinforcement-learning models to dissociate the component processes of learning in the task and test whether the age-related changes observed in choice behavior could be attributed specifically to Pavlovian biases on action-value computation over the course of the learning task. We compared a set of nested reinforcement-learning models that were fit to participants’ data to determine which model best explained choice behavior across all participants. The models were chosen based on prior work in adults using a variant of this task (see Supplementary Table S2 for full list of models tested)^21,33^. Median Akaike Information Criterion (AIC) values, for which lower values reflect a better fit, were used to determine which model provided the best explanation of behavior^37^.

The model that best fit choice behavior included a learning rate, lapse rate, Go bias, Pavlovian bias, and a single reinforcement sensitivity term (Supplementary Table S2; see Methods for details on the other models tested). This model estimated an action-value (Q value or *Q*(*a*,*s*)), for each potential action ($a$; i.e., Go or No-Go) for each of the four robot stimuli (*s*). These Q value estimates were updated on every trial (*t*) using an error-driven learning function (Eq. 1). Outcomes (either 1, 0, -1) were included in the model through the reward term (*r*) and multiplied by a reinforcement sensitivity parameter (*ρ*) that scaled the effective size of rewards and punishments, with higher values magnifying differences between Q values. The difference between the effective reward on that trial (*ρr*_*t*_) and the previous Q value estimate (*Q*_*t-1*_($a$_*t*_,*s*_*t*_)) indexes the reward prediction error, indicating whether the effective outcome was better or worse than expected. The Q value estimate was incrementally updated following each outcome by adding the reward prediction error scaled by a learning rate (*α*).

1 $$Q_{t} \left( {a_{t} ,s_{t} } \right) = Q_{t - 1} (a_{t} ,s_{t} ) + \alpha \left( {\rho r_{t} - Q_{t - 1} (a_{t} ,s_{t} )} \right)$$

Two weights were introduced that altered this action value estimate (weighted action estimate (*W*_*t*_($a$_*t*_,*s*_*t*_))). The first was a Go bias (*b*), which captured an increased tendency to press the button (Eq. 2).

2 $$W_{t} \left( {a,s} \right) = \begin{array}{*{20}l} {Q_{t} \left( {a,s} \right) + b } \hfill & {if\; a = go} \hfill \\ {Q_{t} \left( {a,s} \right)} \hfill & {else} \hfill \\ \end{array}$$

The second was a Pavlovian bias term (*π*) that was multiplied by the stimulus value estimate (*V*_*t*_(*s*)) for each robot (Eq. 3). The Pavlovian bias parameter indexed the degree to which action was facilitated for cues associated with reward and inhibited for those associated with punishment. The stimulus value estimate was updated on each trial through reinforcement, in a similar manner to the Q value estimate (Eq. 4).

3 $$W_{t} \left( {a,s} \right) = \begin{array}{*{20}l} {Q_{t} \left( {a,s} \right) + b + \pi V_{t} \left( s \right)} \hfill & {if\; a = go} \hfill \\ {Q_{t} \left( {a,s} \right)} \hfill & { else} \hfill \\ \end{array}$$

4 $$V_{t} \left( {s_{t} } \right) = V_{t - 1} \left( {s_{t} } \right) + \alpha \left( {\rho r_{t} - V_{t - 1} \left( {s_{t} } \right)} \right)$$

Action values from the model were transformed into choice probabilities using a squashed softmax choice function^38^ that included a lapse rate (*ξ*), which captures the effects of inattention (Eq. 5).

5 $$p(a_{t} |s_{t} ) = \left[ {\frac{{exp(W(a_{t} |s_{t} )}}{{\mathop \sum \nolimits_{a^{\prime}} \exp \left( {W(a^{\prime}|s_{t} )} \right)}}} \right]\left( {1 - \xi } \right) + \frac{\xi }{2}$$

We then investigated patterns of age-related change for each free parameter in the model by performing linear regressions including age alone or both age and age-squared as predictors. Lapse rate, learning rate, and reinforcement sensitivity were best fit by linear regression models that included age alone (Table 1; Supplementary Fig. S3; lapse rate: *F* = 3.192, *p* = 0.079; learning rate: *F* = 0.121, *p* = 0.729; reinforcement sensitivity: *F* = 1.057, *p* = 0.308). However, age was not a significant predictor of lapse rate or learning rate, indicating no evidence of age-related changes in inattention or updating the value of an action (lapse rate: *β* value = − 0.027, s.e. = 0.026, *t*(58) =  − 1.02, *p* = 0.312, Cohen’s *ƒ*^2^ = 0.018; learning rate: *β* value = 0.056, s.e. = 0.033, *t*(58) = 1.672, *p* = 0.1, Cohen’s *ƒ*^2^ = 0.047). There was a significant effect of age on reinforcement sensitivity, revealing greater effective reinforcement with age (*β* value = 0.935, s.e. = 0.396, *t*(58) = 2.362, *p* = 0.022, Cohen’s *ƒ*^2^ = 0.095). The inclusion of age-squared in the model significantly improved the fit for the Go and Pavlovian bias parameters (Go bias: *F* = 4.447, *p* = 0.039; Pavlovian bias: *F* = 11.916, *p* = 0.001). Age-squared, but not linear age, was a significant predictor of the Go bias (age: *β* value =  − 0.039, s.e. = 0.112, *t* =  − 0.350, *p* = 0.728, Cohen’s *ƒ*^2^ = 0.002; age-squared: *β* value = 0.24, s.e. = 0.114, *t* = 2.109, *p* = 0.039, Cohen’s *ƒ*^2^ = 0.077). The Pavlovian bias significantly decreased linearly with age (*β* value =  − 0.903, s.e. = 0.223, *t* =  − 4.044, *p* = 0.001, Cohen’s *ƒ*^2^ = 0.282) and also exhibited a significant effect of age-squared (*β* value = 0.784, s.e. = 0.227, *t* = 3.452, *p* = 0.001, Cohen’s *ƒ*^2^ = 0.205). These nonlinear effects revealed that the Go bias and the Pavlovian bias parameter estimates were both attenuated in adolescents, relative to younger and older individuals. The significant linear relationship between Pavlovian bias and age suggests that, in addition to an adolescent-specific effect, this bias is highest in the youngest individuals and decreases with age. By using the parameter estimates from all compared models to simulate choice behavior for each participant, the best-fitting model qualitatively reproduced the pattern of behavior evident in Fig. 2b (see Supplementary Fig. S4 for predictions from all models). Although parameters from the best-fitting model exhibited some degree of correlation (Supplementary Table S3), parameter recovery revealed that all parameters were highly recoverable (see Parameter Recovery section of the supplement).

**Table 1:** Reinforcement learning parameter estimates.

Parameter	Median (Q1,Q3)			Regression fit: age vs. age^2^
	Child	Adolescent	Adult	
Lapse rate (*ξ*)	0.075 (0.016, 0.250)	0.067 (0.008, 0.229)	0.026 (0.007, 0.139)	Age
Learning rate (*α*)	0.223 (0.055, 0.467)	0.364 (0.173, 0.517)	0.482 (0.164, 0.573)	Age
Go bias (*b*)	0.537 (0.143, 0.942)	0.215 (− 0.007, 0.491)	0.310 (0.006, 1.04)	Age^2^
Pav bias (*π*)	1.388 (0.502, 2.694)	0.229 (0.167, 0.411)	0.398 (0.113, 0.862)	Age^2^
Reinforcement Sensitivity (*ρ*)	3.159 (1.750, 4.247)	4.846 (3.835, 8.383)	4.512 (2.329, 8.758)	Age

Median parameter estimates, as well as the first (Q1) and third (Q3) quartile, are shown separately for each categorical age group. Linear regressions were performed to test the relationship of each parameter estimates with age, which was included as a continuous variable. The addition of age-squared was compared against a model including age alone to identify the best-fitting model, which is listed in the column on the right.

Finally, analysis of response times revealed that older participants made faster responses, and responses to reward-associated stimuli were faster when the correct response was “Go” (Supplementary Table S4).

## Discussion

In this study, participants were tasked with learning the optimal response to cues that were associated with either positive or negative outcomes. We investigated how hard-wired tendencies to approach cues associated with reward and to withhold action to cues associated with punishment might differentially constrain participants’ ability to learn optimal actions over the course of development. Unexpectedly, we found that adolescents’ learning was less biased, relative to both younger and older individuals, in two distinct ways. Whereas children and, to a lesser extent, adults exhibited a robust Pavlovian bias on their action value learning, this interference was reduced in adolescents. Adolescents also displayed a diminished bias toward action over inaction (the “Go” bias).

Adolescents in our study showed better performance in the “No-Go to Win” condition, relative to both adults and children. Consistent with our findings, past work in adults using variants of this task has observed the greatest evidence of Pavlovian interference with instrumental learning in this condition, with performance typically approaching chance in this condition^18,21,39^. Better performance on “Go to Win” than “No-Go to Win” trials can arise not only through reward invigoration of action, but also from an enhanced ability to “assign credit” for rewarding outcomes to past actions, rather than to inaction. Previous studies using task designs capable of dissociating these features of learning find that adults exhibit both a Pavlovian bias on action learning, as well as difficulty linking reward with inaction^39,40^. Thus, it is possible that in addition to a reduced Pavlovian bias, adolescents might also exhibit a heightened ability to learn associations between rewards and past inaction. Future studies should clarify whether developmental differences in the ability to assign credit to passive responses might contribute to adolescents’ superior performance in this task.

Children and adults in our sample, but not adolescents, exhibited a robust Pavlovian bias that was, in part, driven by a suppression of action to punishment-associated cues. In environments in which outcomes are stochastic, actions that are beneficial, on average, may yield occasional negative outcomes. Strong Pavlovian biases may prevent the continued engagement with previously punished contexts that is necessary to discover the opportunities for reward that they present^41–43^. Such tendencies to reflexively withdraw from or avoid situations associated with negative outcomes represent a type of “learning trap” that hinders an individual from sampling sufficiently to discern the true statistics of an environment, allowing potential rewards to go undiscovered^44,45^. Given the many novel contexts that adolescents encounter during their transition toward parental independence^46,47^, a reduced Pavlovian bias might facilitate exploration and unbiased evaluation of action values, allowing adolescents to approach uncertain or ambiguous situations to discover their true value^48^. However, such willingness to continue sampling in stochastic environments that may yield negative outcomes may contribute to a seemingly heightened willingness to take risks during this developmental stage^49,50^.

Pavlovian responses serve critical survival functions^51,52^. However, their expression can also constrain the flexibility of learning. Theoretical accounts propose that the degree of control afforded in learning environments may be used to optimally calibrate the expression of Pavlovian versus instrumental responses^53–56^. Instrumental learning is only adaptive in controllable environments, in which actions can leverage the true contingent causal structure of the environment to bring about beneficial outcomes. In contexts where outcomes are not contingent, the latent assumptions of causality inherent in instrumental learning are overly complex and inaccurate^57^, and reliance on Pavlovian “default” response tendencies may instead be optimal. In active avoidance paradigms, which afford the opportunity for instrumental control of outcomes, adolescent rodents learn to proactively prevent punishment (e.g., by shuttling) better than younger and older animals, whose performance is impaired to a greater degree by the competing Pavlovian tendency to freeze in the face of threat^15,58^. However, in uncontrollable environments, in which there is no effective instrumental response to an anticipated threat, adolescents readily learn and express Pavlovian responses that are particularly resistant to subsequent extinction^59,60^. A parsimonious account of these seemingly inconsistent findings may be that adolescents are particularly effective at detecting the controllability of a given situation and calibrating their reliance on Pavlovian versus instrumental action accordingly. Our finding of an adolescent-specific reduction in Pavlovian bias accords with this explanation, as adolescents might infer the highly controllable nature of our task and adaptively deploy instrumental action. However, further studies are needed to clarify whether the degree of environmental controllability modulates the expression of motivated behaviors differentially across development.

Studies examining the neural substrates of Pavlovian bias in adults suggest how age-related changes in the brain might give rise to the developmental pattern of behavior observed here. Consistent with computational models proposing that the value of both stimuli and actions are evaluated during learning^61–63^, the ventral and dorsal striatum, respectively, encode signals consistent with these computations^64^, and facilitate Pavlovian and instrumental behaviors^65,66^. The prefrontal cortex, through its projections to and from the striatum, is proposed to exhibit sensitivity to contexts in which stimulus and action values conflict and to enable attenuation of Pavlovian reactive biases in such contexts^18,21,39^. Corticostriatal circuitry undergoes marked structural and functional age-related changes from childhood into adulthood^25,32,67–74^. Reduced corticostriatal connectivity in children might constrain their ability to modulate Pavlovian biases on action learning, leading to the heightened expression of valence-action coupling in our youngest participants. Developmental increases in the integration between the prefrontal cortex and striatum may contribute to the linear reductions in these reactive responses with age. Dopamine, a neurotransmitter that innervates both the prefrontal cortex and the striatum, appears to modulate the expression of Pavlovian learning biases^33,40^. The dopaminergic system exhibits substantial reorganization across adolescence^75,76^, including nonlinear changes in dopamine receptor and transporter density^77–81^. Moreover, dopaminergic projections from the striatum to the prefrontal cortex continue to develop in adolescence^82,83^. Such nonlinear changes in the dopaminergic system and corticostriatal connectivity might contribute to the adolescent-specific attenuation of the Pavlovian bias. While the combined influence of these linear and nonlinear changes in corticostriatal circuits and dopaminergic signaling likely contributes to the developmental trajectory of Pavlovian bias expression, future studies are needed to understand the neural mechanisms underlying the age-related pattern observed here.

Although our study focused on interactions between Pavlovian and instrumental learning, an extensive literature further distinguishes two forms of instrumental action that are proposed to reflect distinct underlying computations^84,85^. “Goal-directed” actions are proposed to arise from a “model-based” learning process that computes the value of an action by prospectively searching a mental model of task contingencies and outcomes. In contrast, “habitual” action learning, forgoes the use of a model, instead allowing rewarding outcomes to directly reinforce associations between stimuli and responses. Habitual behavior is well approximated by a “model-free” reinforcement-learning algorithm that incrementally updates a stored estimate of the value of an action. Importantly, these two forms of instrumental action differ in their sensitivity to Pavlovian influence. Pavlovian reactions interfere with habitual behavior to a greater extent than goal-directed action^22,86^. Across tasks that assess these distinct forms of learning, adults who rely more on model-based strategies also exhibit reduced Pavlovian bias^87^. Moreover, pharmacological manipulation of dopamine alters both Pavlovian bias^33,40^ and the use of model-based strategies^88^, suggesting that a common neural mechanism contributes to the expression these distinct forms of value-based learning. While our task cannot directly differentiate goal-directed and habitual forms of instrumental learning, both goal-directed behavior and the model-based evaluations proposed to support it have been found to increase with age across development^25,89–92^. Thus, children’s greater reliance on model-free instrumental learning may confer heightened vulnerability to Pavlovian interference, with a shift toward model-based computation conferring greater resistance to Pavlovian bias with age.

The present study examined the extent to which Pavlovian responses constrain flexible action learning in healthy development. However, strong Pavlovian influences on instrumental action are characteristic of many forms of psychopathology that typically emerge during adolescence^93,94^, including substance abuse and anxiety disorders^95–99^. Adolescence is a period of heightened plasticity in the neural circuits that govern motivated behavior^24,100–103^, which may render adolescents’ expression of Pavlovian action biases particularly sensitive to experiential variation, such as stress exposure^103,104^. Consistent with this notion, past studies in rodents have observed that adolescents’ tendency to imbue cues that predict reward or punishment with inherent motivational value (i.e., “sign-tracking”), is reduced relative to adult animals^105,106^, but that this age-related pattern is reversed when adolescent animals are exposed to adverse rearing environments^107,108^. This experiential sensitivity aligns with evidence in humans that Pavlovian biases on action learning are exacerbated following adolescent exposure to trauma^109^. Further studies examining how life experience interacts with neural plasticity during adolescence to shape learning may shed light on mechanisms that promote resilience or susceptibility to psychopathology.

In this study, we characterized the developmental trajectory of reflexive Pavlovian constraints on flexible action learning. We found that the extent to which these valence-specific response tendencies interfered with instrumental action changed nonlinearly with age, showing a selective attenuation during adolescence. This decreased expression of hard-wired behavioral responses enables greater behavioral flexibility as adolescents learn to respond adaptively to opportunities and challenges in their environment. The influence of Pavlovian learning biases on fundamental behaviors that change over development such as exploration and planning, as well as their mechanistic role in multiple forms of psychopathology that typically emerge prior to adulthood, underscores the importance of a deeper understanding of how interactions between Pavlovian and instrumental learning systems contribute to adaptive and maladaptive behavior over the course of development.

## Methods

### Participants

Previous studies in adults using variants of this task found robust valence-action coupling in sample sizes of 20 participants total^20,21^ or group differences with 20–30 participants per group^110,111^. Thus, we targeted a sample size of 20 participants in each age bin, for a total of 60 participants. This is in line with sample sizes for previous studies that have examined developmental changes in evaluative processes^36,89,92^. Participants were recruited from the New York City metropolitan area through flyers and outreach events. Participants were screened to exclude for a diagnosis of mood or anxiety disorders, a learning disability, current use of beta-blockers or psychoactive medications, or colorblindness. Sixty-two individuals participated in the study. Data from one child were excluded due to a technical error. Our final sample size was 61 participants, consisting of 20 children (8–12 years old, *n* = 10 female, mean age: 10.55, *SD*: 1.34), 20 adolescents (13–17 years old, *n* = 11 female, mean age: 15.38, *SD*: 1.45), and 21 adults (18–25 years old, *n* = 10 female, mean age: 21.28, *SD*: 2.19). The sample included 31 Caucasians (50.82%), 14 individuals of mixed race (22.95%), 12 Asians (19.67%), 3 African Americans (4.92%), and 1 Pacific Islander (1.64%). Eleven participants identified as Hispanic (18.03%). The study protocol was approved by New York University’s Institutional Review Board, the University Committee on Activities Involving Human Subjects. All research was performed in accordance with the relevant guidelines and regulations. All adult participants and parents of minors provided written informed consent and minors provided assent prior to the study.

### Task details

On each trial, participants saw the cue, which was one of the four robots (1,000 ms), followed by a fixation cross (250–3,500 ms), and then the target (described to participants as the robot’s “button”). When participants saw the robot’s “button”, they could decide whether to press the button via a keyboard press (“Go” response) or not press the button (“No-Go” response). Participants had 1,500 ms to respond. If they made a “Go” response, the border of the target would enlarge for the remainder of the 1,500 ms. Following the target, another fixation cross appeared on the screen (1,000 ms) prior to receiving probabilistic feedback (2000 ms). During the inter-trial interval, a fixation cross was presented on the screen (750–1,500 ms).

There were 45 trials for each of the four trial types, resulting in 180 trials total. The colors of the robots were randomized across participants. Stimuli were presented in pseudo-random order ensuring that each robot appeared fifteen times in each of three blocks. After every block of 60 trials, participants were given a break. Prior to the task, participants practiced making “Go” responses by pressing the button and withholding their button press to make “No-Go” responses. Participants also practiced pressing a button and not pressing a button for each type of robot, experiencing that a given robot would give (or take) a ticket for one action and not give (or take) a ticket for the other action. The probabilistic nature of the reinforcements was learned through instruction and experience during the actual game, although no information about the reinforcement probabilities was provided. To encourage learning, participants were instructed that the greater number of tickets won would result in more bonus money at the end of the study, though they were not informed of the specific relationship between tickets and money. In reality, all participants received a $5 bonus regardless of their performance on the task. This experiment was programmed using Cogent 2000, a MATLAB toolbox.

### Statistical analysis

Age and age-squared were z-scored prior to inclusion in any linear regression. To calculate age-squared, age was first z-scored and then squared. All p-values reported within the manuscript reflect a two-tailed significance test unless otherwise indicated. Cohen’s *ƒ*^2^ is a measure of effect size for regression analyses and can be used to calculate the local effect size of predictors in multiple regression. Small, medium, and large effect sizes are represented by *ƒ*^2^ ≥ 0.02, *ƒ*^2^ ≥ 0.15, and *ƒ*^2^ ≥ 0.35, respectively^112^.

### Computational models

Model fitting procedures and parameter estimation were performed using maximum a posteriori method using the fmincon function in Matlab 9.1.0. In addition to the best-fitting model, we also compared models that divided the reinforcement sensitivity term into separate reward sensitivity (*ρ*_*rew*_) and punishment sensitivity (*ρ*_*pun*_) parameters, capturing asymmetry in the effective size of positive and negative reinforcement. Whereas the best-fitting model included weights on the action estimate, in the simplest models the weighted action estimate (*W*_*t*_(*a*_*t*_,*s*_*t*_)) was equivalent to the Q value estimate.

The parameters were constrained as follows: the lapse rate and learning rate between 0 to 1; the reinforcement sensitivity parameters (*ρ*, *ρ*_*rew*_, *ρ*_*pun*_) and the Pavlovian bias between 0 and ∞; the Go bias parameter was not constrained (-∞ to ∞). Priors were chosen to be minimally informative and were based on previous reinforcement learning studies^113^. A prior of *Beta*(1.1, 1.1) was employed for parameters constrained between 0 and 1. A prior of *Normal*(0, 1) was employed for parameters constrained between negative infinity and infinity. A prior of *Gamma*(2, 3) was employed for parameters constrained between 0 and infinity.

## Supplementary information

Supplementary file1.

## Data Availability

Data are available on Open Science Framework: https://osf.io/4h6ne/.

## Code availability

Code to reproduce all analyses in the manuscript can be found on Open Science Framework: https://osf.io/4h6ne/.

## References

[1] Pavlov IP (1927). Conditional reflexes: an investigation of the physiological activity of the cerebral cortex.

[2] Hershberger WA (1986). An approach through the looking-glass. Anim. Learn. Behav..

[3] Williams DR, Williams H (1969). Auto-maintenance in the pigeon: sustained pecking despite contingent non-reinforcement. J. Exp. Anal. Behav..

[4] Gray JA, McNaughton N (2000). The neuropsychology of anxiety: an enquiry into the function of the septo-hippocampal system.

[5] Bolles RC (1970). Species-specific defense reactions and avoidance learning. Psychol. Rev..

[6] O’Doherty, J. P. Multiple systems for the motivational control of behavior and associated neural substrates in humans. In: *Behavioral neuroscience of motivation* (eds. Simpson, E. H. & Balsam, P. D.) 291–312 (Springer International Publishing, Berlin, 2016). 10.1007/7854_2015_386. 10.1007/7854_2015_386 26370947

[7] Boureau Y-L, Dayan P (2011). Opponency revisited: competition and cooperation between dopamine and serotonin. Neuropsychopharmacology.

[8] Breland K, Breland M (1961). The misbehavior of organisms. Am. Psychol..

[9] Dickinson A, Balleine B (1994). Motivational control of goal-directed action. Anim. Learn. Behav..

[10] Estes WK (1943). Discriminative conditioning. I. A discriminative property of conditioned anticipation. J. Exp. Psychol..

[11] Lovibond PF (1983). Facilitation of instrumental behavior by a Pavlovian appetitive conditioned stimulus. J. Exp. Psychol. Anim. Behav. Process..

[12] Rescorla R, Solomon R (1967). Two-process learning theory: relationships between Pavlovian conditioning and instrumental learning. Psychol. Rev..

[13] Choi J-S, Cain CK, LeDoux JE (2010). The role of amygdala nuclei in the expression of auditory signaled two-way active avoidance in rats. Learn. Mem..

[14] Galatzer-Levy IR (2014). Heterogeneity in signaled active avoidance learning: substantive and methodological relevance of diversity in instrumental defensive responses to threat cues. Front. Syst. Neurosci..

[15] Stavnes K, Sprott RL (1975). Effects of age and genotype on acquisition of an active avoidance response in mice. Dev. Psychobiol..

[16] Holland PC (1979). Differential effects of omission contingencies on various components of Pavlovian appetitive conditioned responding in rats. J. Exp. Psychol. Anim. Behav. Process..

[17] Bray S, Rangel A, Shimojo S, Balleine B, O’Doherty JP (2008). The Neural mechanisms underlying the influence of pavlovian cues on human decision making. J. Neurosci..

[18] Cavanagh JF, Eisenberg I, Guitart-Masip M, Huys Q, Frank MJ (2013). Frontal theta overrides pavlovian learning biases. J. Neurosci..

[19] Crockett MJ, Clark L, Robbins TW (2009). Reconciling the role of serotonin in behavioral inhibition and aversion: acute tryptophan depletion abolishes punishment-induced inhibition in humans. J. Neurosci..

[20] Guitart-Masip M (2011). Action dominates valence in anticipatory representations in the human striatum and dopaminergic midbrain. J. Neurosci..

[21] Guitart-Masip M (2012). Go and no-go learning in reward and punishment: interactions between affect and effect. Neuroimage.

[22] Holland PC (2004). Relations between Pavlovian-instrumental transfer and reinforcer devaluation. J. Exp. Psychol. Anim. Behav. Process..

[23] Talmi D, Seymour B, Dayan P, Dolan RJ (2008). Human Pavlovian-instrumental transfer. J. Neurosci..

[24] Hartley CA, Lee FS (2015). Sensitive periods in affective development: nonlinear maturation of fear learning. Neuropsychopharmacology.

[25] Raab, H. A. & Hartley, C. A. The development of goal-directed decision-making. in *Goal-Directed Decision Making* (eds. Morris, R., Bornstein, A. & Shenhav, A.) 279–308 (Academic Press, Cambridge, 2018). 10.1016/B978-0-12-812098-9.00013-9.

[26] Rovee-Collier CK, Gekoski MJ (1979). The economics of infancy: a review of conjugate reinforcement. Adv. Child Dev. Behav..

[27] Shechner T, Hong M, Britton JC, Pine DS, Fox NA (2014). Fear conditioning and extinction across development: evidence from human studies and animal models. Biol. Psychol..

[28] Moutoussis M (2018). Change, stability, and instability in the Pavlovian guidance of behaviour from adolescence to young adulthood. PLOS Comput. Biol..

[29] Bunge SA, Wright SB (2007). Neurodevelopmental changes in working memory and cognitive control. Curr. Opin. Neurobiol..

[30] Diamond, A. The early development of executive functions. in *Lifespan Cognition: Mechanisms of Change. Bialystok E, Craik F, editors.* 70–95 (Oxford University Press, Oxford 2006).

[31] Luna, B. Developmental changes in cognitive control through adolescence. in *Advances in Child Development and Behavior* (ed. Bauer, P.) vol. 37 233–278 (JAI, Amsterdam, 2009). 10.1016/s0065-2407(09)03706-9 PMC2782527 19673164

[32] Somerville LH, Casey B (2010). Developmental neurobiology of cognitive control and motivational systems. Curr. Opin. Neurobiol..

[33] Guitart-Masip M (2014). Differential, but not opponent, effects of L -DOPA and citalopram on action learning with reward and punishment. Psychopharmacology.

[34] Nook EC, Sasse SF, Lambert HK, McLaughlin KA, Somerville LH (2018). The nonlinear development of emotion differentiation: granular emotional experience is low in adolescence. Psychol. Sci..

[35] Rodman AM, Powers KE, Somerville LH (2017). Development of self-protective biases in response to social evaluative feedback. Proc. Natl. Acad. Sci..

[36] Somerville LH (2013). The medial prefrontal cortex and the emergence of self-conscious emotion in adolescence. Psychol. Sci..

[37] Akaike H (1974). A new look at the statistical model identification. IEEE Trans. Autom. Control.

[38] Sutton, R. S. & Barto, A. G. *Reinforcement Learning*. (MIT Press, 1998).

[39] Swart JC (2018). Frontal network dynamics reflect neurocomputational mechanisms for reducing maladaptive biases in motivated action. PLOS Biol..

[40] Swart JC (2017). Catecholaminergic challenge uncovers distinct Pavlovian and instrumental mechanisms of motivated (in) action. eLife.

[41] Huys QJM (2012). Bonsai trees in your head: how the pavlovian system sculpts goal-directed choices by pruning decision trees. PLOS Comput. Biol..

[42] Huys QJM (2015). Interplay of approximate planning strategies. Proc. Natl. Acad. Sci..

[43] Lally N (2017). The neural basis of aversive Pavlovian guidance during planning. J. Neurosci..

[44] Denrell J, March JG (2001). Adaptation as information restriction: the hot stove effect. Organ. Sci..

[45] Rich AS, Gureckis TM (2018). The limits of learning: exploration, generalization, and the development of learning traps. J. Exp. Psychol. Gen..

[46] Casey B, Duhoux S, Cohen MM (2010). Adolescence: what do transmission, transition, and translation have to do with it?. Neuron.

[47] Spear LP (2000). The adolescent brain and age-related behavioral manifestations. Neurosci. Biobehav. Rev..

[48] Tymula A (2012). Adolescents’ risk-taking behavior is driven by tolerance to ambiguity. Proc. Natl. Acad. Sci..

[49] Rosenbaum, G. M. & Hartley, C. A. Developmental perspectives on risky and impulsive choice. *Philos. Trans. R. Soc. B Biol. Sci.* **374**, 20180133 (2019). 10.1098/rstb.2018.0133 PMC6335462 30966918

[50] Steinberg L (2008). A social neuroscience perspective on adolescent risk-taking. Dev. Rev. DR.

[51] Bach DR, Dayan P (2017). Algorithms for survival: a comparative perspective on emotions. Nat. Rev. Neurosci..

[52] LeDoux J, Daw ND (2018). Surviving threats: neural circuit and computational implications of a new taxonomy of defensive behaviour. Nat. Rev. Neurosci..

[53] Huys QJM, Dayan P (2009). A Bayesian formulation of behavioral control. Cognition.

[54] Lloyd, K. & Dayan, P. Safety out of control: dopamine and defence. *Behav. Brain Funct.* **12**, (2016). 10.1186/s12993-016-0099-7 PMC4878001 27216176

[55] Moscarello JM, Hartley CA (2017). Agency and the calibration of motivated behavior. Trends Cogn. Sci..

[56] Rigoli F, Pezzulo G, Dolan RJ (2016). Prospective and Pavlovian mechanisms in aversive behaviour. Cognition.

[57] Dorfman HM, Gershman SJ (2019). Controllability governs the balance between Pavlovian and instrumental action selection. Nat. Commun..

[58] Bauer RH (1978). Ontogeny of two-way avoidance in male and female rats. Dev. Psychobiol..

[59] McCallum J, Kim JH, Richardson R (2010). Impaired extinction retention in adolescent rats: effects of D-Cycloserine. Neuropsychopharmacology.

[60] Pattwell SS (2012). Altered fear learning across development in both mouse and human. Proc. Natl. Acad. Sci..

[61] Barto, A. G. Adaptive critics and the basal ganglia. in *Models of information processing in the basal ganglia* 215–232 (The MIT Press, Cambridge, 1995).

[62] Barto AG, Sutton RS, Anderson CW (1983). Neuronlike adaptive elements that can solve difficult learning control problems. IEEE Trans. Syst. Man Cybern..

[63] Maia TV (2010). Two-factor theory, the actor-critic model, and conditioned avoidance. Learn. Behav..

[64] O’Doherty J (2004). Dissociable roles of ventral and dorsal striatum in instrumental conditioning. Science.

[65] Cardinal RN, Parkinson JA, Hall J, Everitt BJ (2002). Emotion and motivation: the role of the amygdala, ventral striatum, and prefrontal cortex. Neurosci. Biobehav. Rev..

[66] Dayan P, Balleine BW (2002). Reward, motivation, and reinforcement learning. Neuron.

[67] van den Bos W, Cohen MX, Kahnt T, Crone EA (2012). Striatum-medial prefrontal cortex connectivity predicts developmental changes in reinforcement learning. Cereb. Cortex.

[68] van Duijvenvoorde ACK, Achterberg M, Braams BR, Peters S, Crone EA (2016). Testing a dual-systems model of adolescent brain development using resting-state connectivity analyses. NeuroImage.

[69] Gogtay N (2004). Dynamic mapping of human cortical development during childhood through early adulthood. Proc. Natl. Acad. Sci. U. S. A..

[70] Larsen B, Verstynen TD, Yeh F-C, Luna B (2018). Developmental changes in the integration of affective and cognitive corticostriatal pathways are associated with reward-driven behavior. Cereb. Cortex.

[71] Liston C (2006). Frontostriatal microstructure modulates efficient recruitment of cognitive control. Cereb. Cortex N. Y. N.

[72] Mills KL (2016). Structural brain development between childhood and adulthood: convergence across four longitudinal samples. NeuroImage.

[73] Raznahan A (2014). Longitudinal four-dimensional mapping of subcortical anatomy in human development. Proc. Natl. Acad. Sci..

[74] Sowell ER, Thompson PM, Holmes CJ, Jernigan TL, Toga AW (1999). In vivo evidence for post-adolescent brain maturation in frontal and striatal regions. Nat. Neurosci..

[75] Hoops D, Flores C (2017). Making dopamine connections in adolescence. Trends Neurosci..

[76] Wahlstrom D, White T, Luciana M (2010). Neurobehavioral evidence for changes in dopamine system activity during adolescence. Neurosci. Biobehav. Rev..

[77] Andersen SL, Rutstein M, Benzo JM, Hostetter JC, Teicher MH (1997). Sex differences in dopamine receptor overproduction and elimination. NeuroReport.

[78] Andersen SL, Thompson AT, Rutstein M, Hostetter JC, Teicher MH (2000). Dopamine receptor pruning in prefrontal cortex during the periadolescent period in rats. Synapse.

[79] Meng SZ, Ozawa Y, Itoh M, Takashima S (1999). Developmental and age-related changes of dopamine transporter, and dopamine D1 and D2 receptors in human basal ganglia. Brain Res..

[80] Montague DM, Lawler CP, Mailman RB, Gilmore JH (1999). Developmental regulation of the dopamine D 1 receptor in human caudate and putamen. Neuropsychopharmacology.

[81] Teicher MH, Andersen SL, Hostetter JC (1995). Evidence for dopamine receptor pruning between adolescence and adulthood in striatum but not nucleus accumbens. Dev. Brain Res..

[82] Hoops, D., Reynolds, L. M., Restrepo-Lozano, J.-M. & Flores, C. Dopamine development in the mouse orbital prefrontal cortex is protracted and sensitive to amphetamine in adolescence. *eNeuro* **5**, (2018). 10.1523/ENEURO.0372-17.2017 PMC5762649 29333488

[83] Reynolds LM (2018). DCC receptors drive prefrontal cortex maturation by determining dopamine axon targeting in adolescence. Biol. Psychiatry.

[84] Balleine BW, O’Doherty JP (2010). Human and rodent homologies in action control: corticostriatal determinants of goal-directed and habitual action. Neuropsychopharmacology.

[85] Dickinson A (1985). Actions and habits: the development of behavioural autonomy. Phil. Trans. R. Soc. Lond. B.

[86] Yin HH, Knowlton BJ (2006). The role of the basal ganglia in habit formation. Nat. Rev. Neurosci..

[87] Sebold M (2016). Don’t think, just feel the music: individuals with strong pavlovian-to-instrumental transfer effects rely less on model-based reinforcement learning. J. Cogn. Neurosci..

[88] Wunderlich K, Smittenaar P, Dolan RJ (2012). Dopamine enhances model-based over model-free choice behavior. Neuron.

[89] Decker JH, Otto AR, Daw ND, Hartley CA (2016). From creatures of habit to goal-directed learners tracking the developmental emergence of model-based reinforcement learning. Psychol. Sci..

[90] Kenward B, Folke S, Holmberg J, Johansson A, Gredebäck G (2009). Goal directedness and decision making in infants. Dev. Psychol..

[91] Klossek UMH, Russell J, Dickinson A (2008). The control of instrumental action following outcome devaluation in young children aged between 1 and 4 years. J. Exp. Psychol. Gen..

[92] Potter TCS, Bryce NV, Hartley CA (2017). Cognitive components underpinning the development of model-based learning. Dev. Cogn. Neurosci..

[93] Kessler RC (2005). Lifetime prevalence and age-of-onset distributions of dsm-iv disorders in the national comorbidity survey replication. Arch. Gen. Psychiatry.

[94] Paus T, Keshavan M, Giedd JN (2008). Why do many psychiatric disorders emerge during adolescence?. Nat. Rev. Neurosci..

[95] Carter BL, Tiffany ST (1999). Meta-analysis of cue-reactivity in addiction research. Addict. Abingdon Engl..

[96] Everitt BJ, Dickinson A, Robbins TW (2001). The neuropsychological basis of addictive behaviour. Brain Res. Rev..

[97] Garbusow M (2016). Pavlovian-to-instrumental transfer effects in the nucleus accumbens relate to relapse in alcohol dependence. Addict. Biol..

[98] Mineka S, Oehlberg K (2008). The relevance of recent developments in classical conditioning to understanding the etiology and maintenance of anxiety disorders. Acta Psychol. (Amst.).

[99] Mkrtchian A, Aylward J, Dayan P, Roiser JP, Robinson OJ (2017). Modeling avoidance in mood and anxiety disorders using reinforcement learning. Biol. Psychiatry.

[100] Dahl, R. E. Adolescent brain development: a period of vulnerabilities and opportunities. Keynote Address. *Ann. N. Y. Acad. Sci.* **1021**, 1–22 (2004). 10.1196/annals.1308.001 15251869

[101] Fuhrmann D, Knoll LJ, Blakemore S-J (2015). Adolescence as a sensitive period of brain development. Trends Cogn. Sci..

[102] Meyer HC, Lee FS (2019). Translating developmental neuroscience to understand risk for psychiatric disorders. Am. J. Psychiatry.

[103] Romeo RD, McEwen BS (2006). Stress and the adolescent brain. Ann. N. Y. Acad. Sci..

[104] Lupien SJ, McEwen BS, Gunnar MR, Heim C (2009). Effects of stress throughout the lifespan on the brain, behaviour and cognition. Nat. Rev. Neurosci..

[105] Anderson RI, Spear LP (2011). Autoshaping in adolescence enhances sign-tracking behavior in adulthood: Impact on ethanol consumption. Pharmacol. Biochem. Behav..

[106] Doremus-Fitzwater TL, Spear LP (2011). Amphetamine-induced incentive sensitization of sign-tracking behavior in adolescent and adult female rats. Behav. Neurosci..

[107] Anderson RI, Bush PC, Spear LP (2013). Environmental manipulations alter age differences in attribution of incentive salience to reward-paired cues. Behav. Brain Res..

[108] DeAngeli NE, Miller SB, Meyer HC, Bucci DJ (2017). Increased sign-tracking behavior in adolescent rats. Dev. Psychobiol..

[109] Ousdal OT (2018). The impact of traumatic stress on Pavlovian biases. Psychol. Med..

[110] de Boer L (2019). Dorsal striatal dopamine D1 receptor availability predicts an instrumental bias in action learning. Proc. Natl. Acad. Sci..

[111] Guitart-Masip M (2012). Action controls dopaminergic enhancement of reward representations. Proc. Natl. Acad. Sci..

[112] Cohen J (1992). A power primer. Psychol. Bull..

[113] Daw ND, Gershman SJ, Seymour B, Dayan P, Dolan RJ (2011). Model-based influences on humans’ choices and striatal prediction errors. Neuron.

