# Supplementary material for: Adolescents exhibit reduced Pavlovian biases on instrumental learning
Source: Sci Rep. 2020 Sep 25;10:15770. doi: 10.1038/s41598-020-72628-w (PMC7519144; doi:10.1038/s41598-020-72628-w)
Supplement: Supplementary file 1 — Supplementary file1. [file 41598_2020_72628_MOESM1_ESM.pdf]

Supplementary materials

Adolescents exhibit reduced Pavlovian biases on instrumental learning

Hillary A. Raab[1] & Catherine A. Hartley[1,2,*]

[1]Department of Psychology, New York University, New York, NY
[2]Center for Neural Science, New York University, New York, NY

*Correspondence concerning this manuscript should be addressed to Catherine A. Hartley (cate@nyu.edu), Department of Psychology, New York University, 6 Washington Pl., New York, NY 10003.

Supplementary Analyses

Logistic regression

To investigate the contributions of Pavlovian and instrumental learning across development, accuracy for the four different trial types (i.e. Go to Win, Go to Avoid Losing, No-Go to Win, No-Go to Avoid Losing) was assessed. We performed a mixed-effects logistic regression including valence (reward or punishment), action (go or no-go), and age (*z*-score transformed), as independent predictors of correct choice in the model using the lme4 package (Version 1.1-13) for R software [1,2]. The logistic regression was repeated including both age and age-squared (both *z*-score transformed) predictors to test which model provided a better fit. All interactions with age and age-squared were included as fixed-effects. Subject-specific adjustments to the fixed intercept were specified in the model. The terms of interest were the valence-by-action interaction (i.e., the degree of Pavlovian facilitation or interference) and the valence-by-action-by-age interaction (i.e., developmental changes in the interaction between Pavlovian and instrumental behaviors). The inclusion of age-squared as a predictor of correct choice significantly improved the model fit ($f^2(4,13) = 39.464$, $p = 5.59 \times 10^{-8}$). Participants' accuracy was greater when Pavlovian reactive biases were aligned with correct instrumental responses, compared to when they were in conflict (valence-by-action interaction; Supplementary Table S1). This Pavlovian bias effect also interacted with age and age-squared (valence-by-action-by-age and valence-by-action-by-age-squared interactions; Supplementary Table S1.), revealing differential Pavlovian influences on instrumental action across development.

Response time analyses

We examined whether responses were faster when Pavlovian expectancies were aligned with instrumental contingencies to press a button as compared to in opposition. All "Go" responses regardless of the accuracy were log-transformed prior to being included in linear regressions. Similar to the logistic regression of correct choice, the response time analysis included cue valence (reward or punishment), correct action (go or no-go), and age (z-score transformed) as independent predictors in the model using lme4 package (Version 1.1-13) for R software [1,2]. Subject-specific adjustments to the fixed intercept were specified in the model. A linear model that included age as a predictor of response time significantly improved the fit compared to a model without the inclusion of an age term ($f^2(4) = 20.06$, $p = .0005$). Participants were faster to respond to reward-associated stimuli, when the correct response was "Go," and with increasing age (Supplementary Table S4).

Correlation between Pavlovian parameter estimates and Pavlovian performance bias values

To ensure that the Pavlovian parameter values estimated from the computational model are aligned with the Pavlovian performance bias values, we performed a non-parametric Spearman's rank correlation between the two variables. As we hypothesized a positive relationship between the two, we used a one-sided test. As expected, this analysis revealed a robust positive correlation between the two distinct ways of calculating Pavlovian bias ($\rho(59) = .531$, $p < .001$).

Relationship between Reward Invigoration and Punishment Suppression

The Pavlovian performance bias reflects both a reward-based invigoration of action and a punishment-based suppression of action. To confirm that the Pavlovian performance bias was being driven by reflexive responses to both reward and punishment, we performed a Pearson correlation between the reward-based invigoration score and the punishment-based suppression score. This analysis revealed a strong positive correlation between the degree to which reward invigorates action and punishment suppresses action (Supplementary Figure S2; $r = .753$, $t(59)$: 8.794, $p = 2.522e-12$). This relationship remained significant even after performing

a partial correlation to control for age ($r = .763$, $t(59) = 8.983$, $p = 1.414e-12$), suggesting that the relationship between these components of Pavlovian bias does not exhibit age-related change.

Posterior Predictions

Data simulated from each model were used to predict accuracy on the task. Individual subject parameter estimates from each model were used to simulate the choice data, from which accuracy was calculated for each age group and trial type. Model 8, which provided the best fit and includes Go and Pavlovian biases as well as a reinforcement sensitivity parameter, closely approximated participant behavior (Supplementary Figure S4).

Parameter Recovery

As the free parameters from the best-fitting model exhibited some degree of covariance between parameters (Supplementary Table S3), we sought to test if these parameters were recoverable. Parameter recovery was performed by simulating 10,000 sets of parameter values for the best-fitting model (Model 8) by bootstrapping the parameter estimates that were derived from participant choice behavior. These parameters were used to generate choice data that were then fit using Model 8 to see how well the simulated parameters could be recovered. We used Spearman correlation to assess the relation between the simulated and recovered values. All parameters could be recovered well, reflected in the high correlation coefficients (learning rate: $\rho(9998) = .774$; lapse rate $\rho(9998) = .718$; Go bias $\rho(9998) = .811$; Pavlovian bias $\rho(9998) = .773$; Reinforcement sensitivity term $\rho(9998) = .661$; all p's < .001).

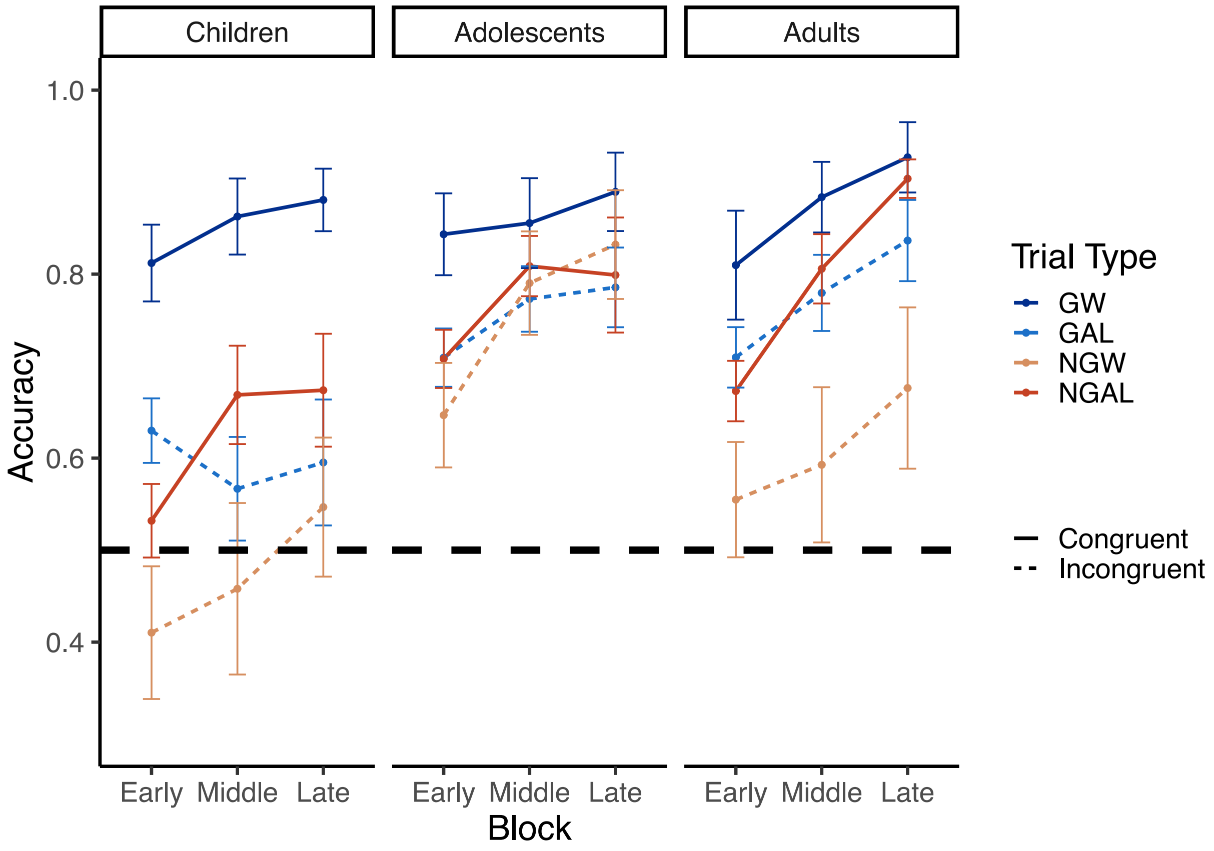

Supplementary Figure 1. Learning curves by age. Accuracy on each trial type is depicted for the early, middle, and late block of the task and separately for children, adolescents, and adults. The darker lines represent when Pavlovian tendencies and correct instrumental responses are aligned where the lighter dashed lines represent when they conflict. The following abbreviations are used: GW: Go to Win; GAL: Go to Avoid Losing; NGW: No-Go to Win; NGAL: No-Go to Avoid Losing. Error bars represent +1 SEM.

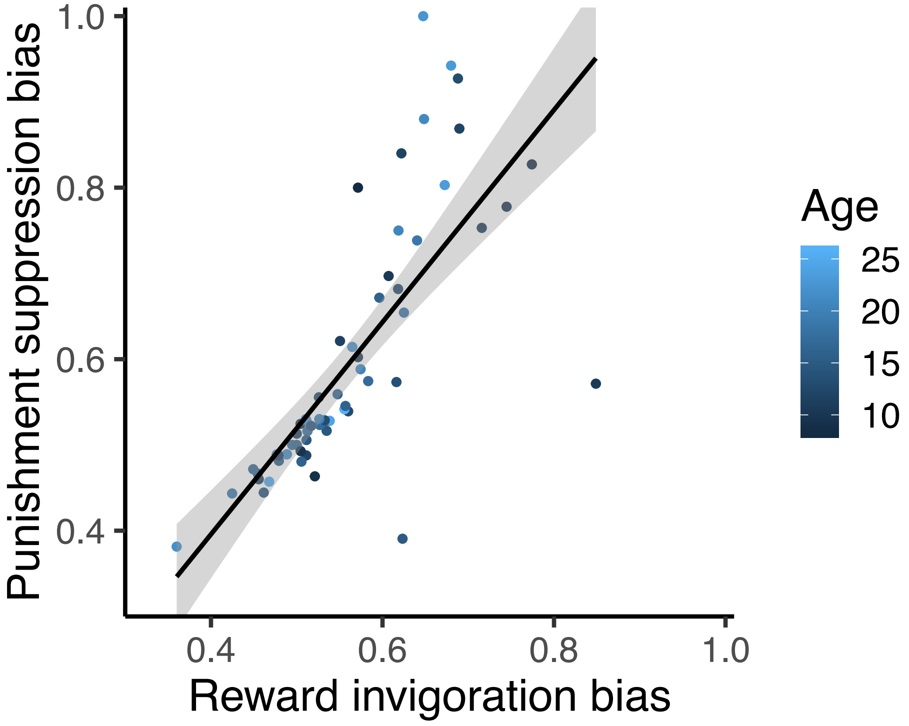

Supplementary Figure 2. Relationship between the two components of the Pavlovian performance bias score (i.e., reward invigoration of action and punishment suppression of action). Age is represented by the color of the data point, with younger individuals depicted in darker colors. The error bars represent a .95 confidence interval.

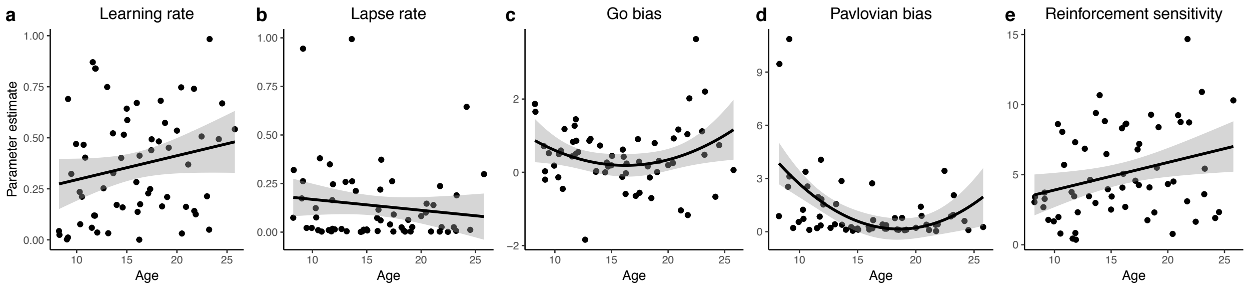

Supplementary Figure 3. The effect of age on parameter estimates from the best fitting model. (a) Learning rate, (b) lapse rate, (c) Go bias, (d) Pavlovian bias, and (e) reinforcement sensitivity are plotted as a function of age. The line of best fit reflects whether the data were best fit by a regression that included age alone or the addition of an age-squared term. The error bars represent a .95 confidence interval.

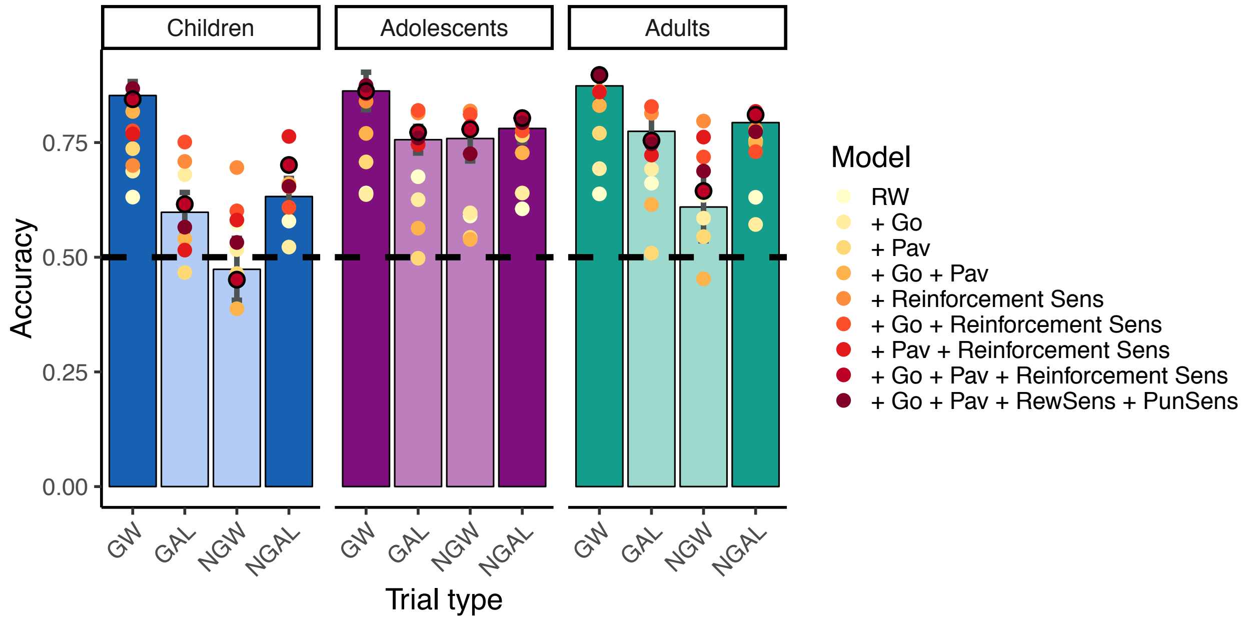

Supplementary Figure 4. Model predictions generated from using parameter values estimated from fitting participants' choice data. The best fitting model (Model 8), which includes Go and Pavlovian biases as well as a reinforcement sensitivity term, is outlined in black.

Supplementary Table 1. Mixed effects logistic regression coefficients showing the effects of valence, action, and valence-by-action interaction with age and age-squared (both continuous variables).

Predictor	Regression Coefficient (*SE*)	*Z*-value	*p*
**Intercept**	**1.534 (0.138)**	**11.133**	**$< 2 \times 10^{-16}$**
**Valence**	**-0.096 (0.037)**	**-2.596**	**0.009**
**Action**	**-0.241 (0.037)**	**-6.492**	**8.47×10^{-11}**
**Age**	**0.355 (0.098)**	**3.607**	**0.0003**
**Age^2**	**-0.329 (0.100)**	**-3.270**	**0.001**
**Valence x Action**	**0.308 (0.037)**	**8.313**	**$< 2 \times 10^{-16}$**
**Valence x Age**	**0.078 (0.024)**	**3.187**	**0.001**
Valence x Age^2	0.011 (0.026)	0.431	0.667
**Action x Age**	**0.048 (0.024)**	**1.983**	**0.047**
**Action x Age^2**	**-0.095 (0.026)**	**-3.631**	**0.00028**
**Valence x Action x Age**	**-0.060 (0.024)**	**-2.472**	**0.013**
**Valence x Action x Age^2**	**0.099 (0.026)**	**3.773**	**0.00016**

Supplementary Table 2. The median Akaike information criterion (AIC) values used to compare model fits. Lower values indicate a better fit.

AIC Model Comparison	
Model	Value
M1: LR + Lapse	199.115
M2: LR + Lapse + Go	191.723
M3: LR + Lapse + Pav	187.646
M4: LR + Lapse + Go + Pav	175.294
M5: LR + Lapse + ReinforcSens	172.75
M6: LR + Lapse + Go + ReinforcSens	162.263
M7: LR + Lapse + Pav + ReinforcSens	167.967
**M8: LR + Lapse + Go + Pav + ReinforcSens**	**157.24**
M9: LR + Lapse + Go + Pav + RewSens + PunSens	157.692

Abbreviations: LR (learning rate, α), Lapse (lapse rate, ξ), Go (Go bias, *b*), Pav (Pavlovian bias, π), ReinforcSens (reinforcement sensitivity, ρ), RewSens (reward sensitivity, ρ_{rew}), PunSens (punishment sensitivity, ρ_{pun})

Supplementary Table 3. The relationship between parameters estimated for all participants from the best-fitting computational model, as measured by Spearman correlations and associated *p*-values.

	Learning rate	Lapse rate	Go bias	Pavlovian bias
Learning rate	-	-	-	-
Lapse rate	.03, *p* =.799	-	-	-
Go bias	-.02, *p* =.852	-.20, *p* = .130	-	-
Pavlovian bias	-.09, *p* = .512	**.35, *p* = .006**	**.29, *p* = .021**	-
Reinforcement sensitivity	**-.27, *p* = .036**	.02, *p* = .864	**-.28, *p* =.032**	**-.48, *p* < .001**

Supplementary Table 4. Linear regression coefficients showing the effects of valence, action, and age on response time.

Predictor	Regression Coefficient (*SE*)	*t*-value	*p*
**Intercept**	**6.213 (0.020)**	**304.767**	**< 2 x 10^{-16}**
**Valence**	**0.027 (0.006)**	**4.252**	**2.16 x 10^{-5}**
**Action**	**0.031 (0.006)**	**4.779**	**1.81 x 10^{-6}**
**Age**	**-0.094 (0.020)**	**-4.593**	**2.2 x 10^{-5}**
Valence x Action	0.002 (0.006)	0.248	0.804
Valence x Age	0.006 (0.006)	0.911	0.362
Action x Age	0.002 (0.006)	0.291	0.771
Valence x Action x Age	-0.001 (0.006)	-0.215	0.830

References

1. Bates, D., Mächler, M., Bolker, B. & Walker, S. *lme4: Linear Mixed-Effects Models using ‘Eigen’ and S4*. (2015).
2. R Core Team. *R: A language and environment for statistical computing.* (R Foundation for Statistical Computing, 2017).
